# Challenging Endocrine Sensitivity of Hormone Receptor-Positive/HER2-Negative Advanced Breast Cancer with the Combination of Eribulin and Endocrine Therapy: The REVERT Study

**DOI:** 10.3390/cancers14235880

**Published:** 2022-11-29

**Authors:** Ana López González, Sonia Del Barco Berrón, Isabel Grau, Maria Galan, Beatriz Castelo Fernández, Alfonso Cortés, Pedro Sánchez Rovira, Alejandro Martinez-Bueno, Xavier Gonzalez, Almudena García, Petra Gener, Leonardo Mina, Daniel Alcalá-López, Miguel Sampayo, Javier Cortés, José Manuel Pérez-Garcia, Antonio Llombart-Cussac, Elena López-Miranda

**Affiliations:** 1 Complejo Asistencial Universitario de León, 24071 León, Spain; 2 Institut Català d’Oncologia, 17007 Girona, Spain; 3 Hospital Son Llatzer, 7198 Palma, Spain; 4 Hospital Universitario La Paz, 28046 Madrid, Spain; 5 Hospital Universitario Ramón y Cajal, 2559 Madrid, Spain; 6 Complejo Hospitalario Ciudad de Jaén, 23007 Jaén, Spain; 7 Insituto Oncológico Dr. Rosell, Hospital Quiron Dexeus, 08028 Barcelona, Spain; 8 Instituto Oncológico Dr. Rosell, Hospital General de Cataluña, 08190 San Cugat del Vallés, Spain; 9 International Breast Cancer Center (IBCC), Pangaea Oncology, Quiron Group, 08017 Barcelona, Spain; 10 Medica Scientia Innovation Research SL (MEDSIR), 08018 Barcelona, Spain; 11 Department of Medicine, Faculty of Biomedical and Health Sciences, Universidad Europea de Madrid, 28670 Madrid, Spain; 12 Arnau de Vilanova Hospital, Universidad Católica de Valencia, 46015 Valencia, Spain

**Keywords:** eribulin, luminal breast cancer, endocrine resistance

## Abstract

**Simple Summary:**

Acquiring of resistance is a common outcome after prolonged cancer treatment and consecutive treatments are, in general terms, limited. Here we published the results of REVERT clinical study that aimed to REVERT the resistance to hormonal treatment that often occurs in breast cancer patients positive for hormone receptors. According to previous published data, adding drug called eribulin to hormonal treatment may sensitized the tumor to the hormonal treatment due to switch of cancer cell phenotype. Even though this theory was not proved in this study, the outcomes of this study open a new door for further investigation since the results suggests that the patients treated with hormonal therapy and inhibitors of cyclins may have further clinical benefit, if eribulin is added to the therapy. Importantly, no additional unexpected side effects were reported with this drug combination.

**Abstract:**

Background: Luminal advanced breast cancer (ABC) patients eventually progress on endocrine therapy. REVERT aimed to explore whether eribulin could restore endocrine sensitivity in a randomized, non-comparative phase II trial. Methods: Aromatase inhibitor (AI)-resistant patients with luminal ABC were randomized 1:1 to receive eribulin +/− AI. Patients were stratified by prior cyclin-dependent kinases 4/6 inhibitor (CDK4/6i) treatment. The primary endpoint was an investigator-assessed overall response rate (ORR) according to RECIST version 1.1 in the eribulin + AI arm. An interim analysis was planned with 11 evaluable patients according to a two-stage Simon design. Results: Twenty-two patients were enrolled (15 eribulin + AI arm; 7 eribulin arm). The trial was terminated early in March 2021, with eight (36.4%) patients still on treatment. ORR was 26.7% in the eribulin + AI arm (95% CI, 7.8–55.1%; *p* = 0.0541). In the eribulin arm, two (28.6%) patients had an objective response (95% CI, 3.7–71.0%). The difference between the study arms was not significant (*p* = 0.918). The addition of AI to eribulin also failed to show improvement in other efficacy endpoints. A significant interaction between the treatment arm and previous CDK4/6i treatment was observed for ORR (*p* = 0.018) and progression-free survival (*p* = 0.084). Overall, the toxicity profile was consistent with the known safety profile of eribulin. No treatment-related deaths were reported. Conclusion: Eribulin + AI does not seem to improve outcomes compared with eribulin monotherapy in patients with AI-resistant luminal ABC. This chemo–endocrine approach deserves further investigation after progression to CDK4/6i-based therapy.

## 1. Introduction

Luminal breast cancer, defined as hormone receptor-positive (HR[+])/human epidermal growth factor receptor 2-negative (HER2[−]) tumors, is the most common (~65%) subtype in postmenopausal patients with advanced breast cancer (ABC). Endocrine therapy (ET) is the mainstay of treatment for these patients. The third-generation aromatase inhibitors (AI) (letrozole, anastrozole, and exemestane) for first-line, and fulvestrant for second-line treatment, have been the standard therapy for these patients over the last few years [1,2,3]. The prognosis of luminal ABC patients has been further substantially improved with the addition of cyclin-dependent kinases 4 and 6 (CDK4/6) inhibitors (CDK4/6i) to ET [4]. Unfortunately, most patients eventually progress on ET [2]. Therefore, the treatment of these patients and the clinical development of strategies that restore sensitivity to ET represent an unmet medical need.

Eribulin is an antineoplastic agent that inhibits microtubule dynamics [5,6]. Interestingly, eribulin also exhibits non-mitotic activity including antiangiogenetic action, the capacity to suppress epithelial–mesenchymal transition (EMT), and the ability to inhibit migration and invasion of cancer cells and metastasis [7,8].

Currently, eribulin is approved as a monotherapy for the treatment of patients with ABC who have previously received at least one chemotherapeutic regimen for advanced disease, including a taxane and an anthracycline in either the adjuvant or metastatic setting, unless patients were not suitable for these treatments. The approval of eribulin was based on the results of the 305/EMBRACE randomized phase III trial, in which treatment with eribulin conferred an overall survival (OS) benefit of 2.5 months over treatment of the physician’s choice in women with ABC previously treated with two to five lines of chemotherapy in the metastatic setting [9]. Its efficacy in ABC was further supported by the 301 study that showed similar efficacy of eribulin and capecitabine in terms of OS and progression-free survival (PFS) in patients who had received up to two prior chemotherapy regimens for advanced disease [10,11].

Furthermore, in line with its reversion potential, neoadjuvant treatment with eribulin changed the phenotype of 50% of aggressive luminal B tumors, inducing phenotypical changes consistent with the luminal A molecular subtype in the NeoEribulin-SOLTI1007 trial [12]. In consequence, we hypothesized that eribulin could convert more aggressive tumors into less aggressive tumors with higher sensitivity to ET.

The objective of the REVERT trial was to test whether a combination of eribulin and AI could have synergistic activity due to a potential restoration of endocrine sensitivity. In this trial, we explored the efficacy and safety of the eribulin + AI combination in HR[+]/HER2[-] ABC patients with well-established AI resistance criteria and no prior chemotherapy for metastatic disease.

## 2. Material and Methods

### 2.1. Study Design and Patients

REVERT (NCT03795012) was a multicenter, randomized, non-comparative phase II trial of eribulin + AI or eribulin in women with AI-resistant HR[+]/HER2[−] ABC. REVERT was conducted in seven centers in Spain.

Eligible patients were postmenopausal women aged ≥18 years with HR[+]/HER2[−] ABC whose disease had progressed while on an AI-containing regimen in the metastatic setting but not necessarily in the treatment line immediately before, or who had relapsed during the adjuvant treatment with an AI or within six months after its completion. Patients must have received an anthracycline- and/or taxane-based regimen in the (neo)adjuvant setting and up to three prior lines of ET for ABC, except for those patients who had relapsed during the adjuvant treatment.

Patients must have measurable disease (according to Response Evaluation Criteria in Solid Tumors RECIST version 1.1), an Eastern Cooperative Oncology Group (ECOG) performance status of 0–1, and adequate hematological counts and hepatic and renal function.

Key exclusion criteria included previous chemotherapy for ABC and known uncontrolled or progressive central nervous system metastases. Full eligibility criteria are described in the study protocol (Supplemental material).

The study protocol and supporting documents were approved by the institutional review board at each site. All patients provided written informed consent prior to participation in any study-related activities. This study was performed in accordance with ethical principles consistent with the Declaration of Helsinki and the International Council of Harmonization/Good Clinical Practice as well as all applicable regulatory requirements.

### 2.2. Randomization and Masking

Patients were randomly assigned in a 1:1 ratio to receive eribulin + AI or eribulin monotherapy. A central block randomization procedure with a block size of four was set up with the web-based software OpenClinica, version 3.14 (https://www.openclinica.com/, accessed on 12 February 2022). Randomization was stratified according to previous use of CDK4/6i. An independent biometrical company (SAIL Biometría) developed the sequence generation and allocation concealment. The recruitment, selection, and treatment procedures were conducted by investigators and site staff. All study participants were aware of their treatment assignment.

### 2.3. Treatment

Patients received 1.23 mg/m^2^ eribulin intravenously on days 1 and 8 of every 21-day cycle, alone or in combination with an AI (anastrozole [1 mg], letrozole [2.5 mg], or exemestane [25 mg] to be taken once daily). The AI had to be identical to the last AI administered to the patient in either the adjuvant or metastatic setting. Premenopausal women also received concomitant treatment with luteinizing hormone-releasing hormone (LHRH) analogs. Patients received treatment until disease progression, unacceptable toxicity, death, or discontinuation from the study treatment for any other reason.

Dose interruption and reductions were allowed for eribulin, as defined by prespecified guidelines in the protocol but were not applicable for ET.

### 2.4. Study Assessments

Study visits occurred on day 1 of each 21-day cycle, with a safety follow-up visit within 30 days after treatment discontinuation, and survival follow-up visits every three months until the end of the study thereafter.

Tumor assessments were carried out by computed tomography or magnetic resonance imaging according to RECIST version 1.1 at baseline and every nine weeks until disease progression, initiation of a new anticancer therapy, or withdrawal from the study, whichever came first. Bone scans were performed every 27 weeks for patients with bone lesions identified at baseline unless there was clinically or biochemically suspected bone progression.

Laboratory tests were carried out on day 1 of every cycle and according to local standard treatment and clinical indications before treatment administration. Vital signs, weight, and ECOG performance status were assessed on day 1 of every cycle.

Safety was evaluated on days 1 and 8 of every cycle in all patients who had received at least one dose of study treatment by assessment of adverse events (AEs), clinical laboratory tests, physical examinations, and vital signs.

### 2.5. Study Endpoints

The primary efficacy endpoint was an overall response rate (ORR) according to RECIST version 1.1 as assessed by an investigator review in patients treated with the combination of eribulin + AI. The ORR was defined as the best overall response of either a complete response or partial response. Tumor response had to be confirmed after four weeks, as per RECIST version 1.1.

Secondary endpoints included the ORR in patients treated with eribulin monotherapy, other efficacy objectives in both arms (PFS, clinical benefit rate [CBR], duration of response [DoR], time to response [TTR], maximum tumor shrinkage, and OS), and safety determined by the National Cancer Institute (NCI) Common Terminology Criteria for Adverse Events (CTCAE) version 5.0.

### 2.6. Sample Size Calculation and Statistical Analysis

The primary endpoint was the proportion of patients who achieved an objective response in the eribulin + AI treatment arm. We planned to assign 30 patients to receive eribulin + AI and 30 patients to receive eribulin monotherapy (N = 60). The protocol specified one interim analysis for the eribulin + AI treatment arm with 11 evaluable patients, based on a Simon’s admissible two-stage design. The study would continue with the second stage if ≥2 responders were observed in the eribulin + AI treatment arm. The enrollment continued during the interim data analysis. The critical value for the final analysis in this arm was ≥6 patients with an objective response among 27 patients. The objective response analysis was designed to test the null hypothesis that the true ORR was ≤10%. The alternative hypothesis was that the true ORR was ≥30%. Considering a dropout rate of 10%, a sample size of 30 patients was needed to attain 80% power at a nominal one-sided alpha level of 0.05.

ORR, PFS, CBR, maximum tumor shrinkage, and OS were analyzed in the intention-to-treat population, defined as all randomized patients. TTR and DoR were analyzed in all patients who had an objective response, which included either a complete or partial response. Safety was assessed in all patients who had received at least one dose of study treatment.

ORR and CBR were estimated with Clopper–Pearson 95% confidence intervals (CI). Treatment differences in objective response and clinical benefit were assessed using the Wald test stratified by the previous use of CDK4/6i in a logistic regression model. PFS and OS were estimated using the Kaplan–Meier method. Treatment differences in PFS and OS were assessed using the Wald test stratified by the previous use of CDK4/6i in Cox proportion hazard models with Efron’s method of tie handling. Treatment differences in DoR and TTR were determined using the Wilcoxon test. The maximum tumor shrinkage was described in accordance with the best response and the study arm with waterfall plots.

The consistency-of-treatment effect was assessed across patients with or without previous treatment with CDK4/6i. It was tested in a logistic model for an objective response or a Cox model for PFS with a treatment-by-factor interaction term. The analyses were conducted with a model-based likelihood ratio test set at a 2-sided 0.1 α level.

For all secondary endpoints, we used two-sided *p*-values with an alpha ≤0.05 level of significance and 95% CI. However, the trial was terminated early and did not meet its primary objective. The *p*-values and 95% CI are descriptive and should not be interpreted as a measure of statistical relevance. They are reported as a rough guide to plan new hypothesis-driven experiments and inform about the uncertainty of our exploratory data.

## 3. Results

### 3.1. Patient Disposition and Interim Analysis

**Figure 1:**
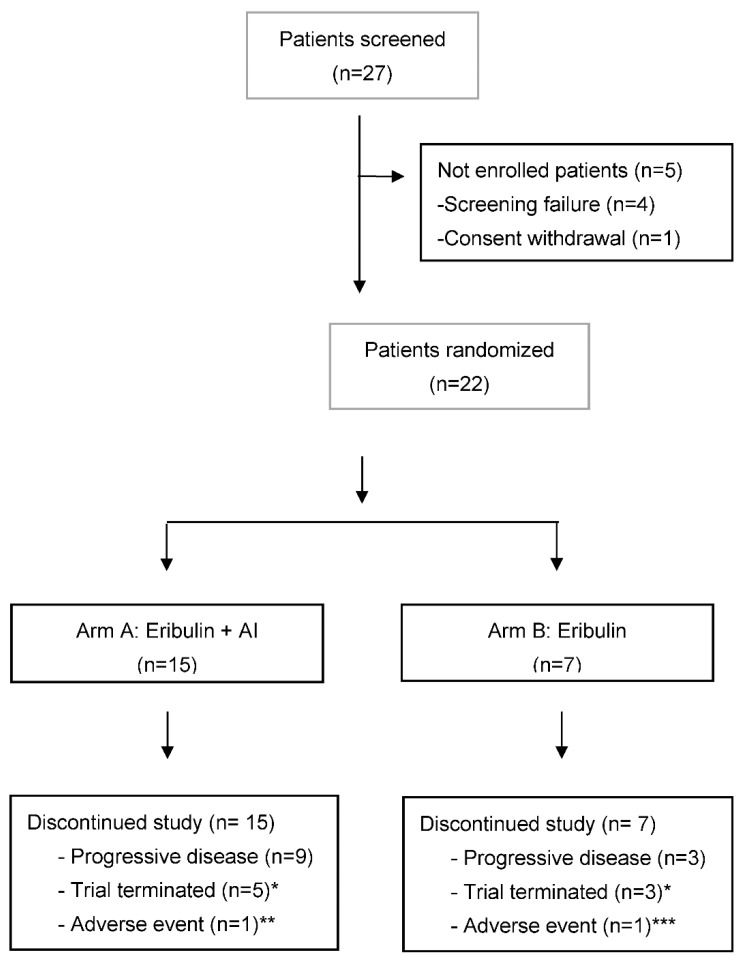
Patient disposition. * Patients continued the treatment outside the clinical trial. ** Grade 3 peripheral neuropathy related to eribulin. *** Grade 3 pneumonitis unrelated to study treatment.

Between June 2019 and January 2021, a total of 15 (55.6%) patients were enrolled in the eribulin + AI arm, and seven (25.9%) patients were enrolled in the eribulin monotherapy arm. All randomized patients received at least one dose of study treatment (Figure 1).

On 14 January 2021, the REVERT data monitoring committee with input from the funder, on review of the interim analysis, determined that there was not enough evidence to support continued accrual.

### 3.2. Patient Characteristics

Patient characteristics were well balanced between treatment arms. Among all randomized patients, the median age was 65 (range 56–77) years and the ECOG performance score was 0 in 18 (81.8%) patients. All patients had visceral disease, 14 (63.6%) patients had liver involvement, and eight (36.4%) patients presented ≥3 metastatic sites. A total of nine (45.5%) patients had not received ET for metastatic disease. In terms of the last AI that patients had previously received, 15 (68.2%) patients received letrozole, six (27.3%) patients received exemestane, and one (4.5%) patient received anastrozole. Twelve (54.5%) patients were previously treated with CDK4/6i, and nine (40.9%) patients received the CDK4/6i in the immediate previous line of therapy. The overall demographic and baseline characteristic data for all patients are listed in Table 1.

### 3.3. Treatment

At the time of the data cutoff (31 March 2021), five (33.3%) patients receiving eribulin + AI and three (42.9%) patients treated with eribulin monotherapy continued the treatment outside of the clinical trial. Treatment discontinuation was primarily due to disease progression, which occurred in nine (60.0%) patients in the eribulin + AI arm and three (42.9%) patients in the eribulin arm (Figure 1).

The median duration of eribulin treatment was 4.4 months (interquartile range [IQR], 2.3–6.7 months) for the eribulin + AI arm and 4.9 months (IQR, 3.8–7.4 months) for the eribulin arm. The median relative dose intensity of eribulin was 91.2% (IQR, 81.3–95.8%) for the eribulin + AI arm and 96.3% (IQR, 89.9–98.8%) for the eribulin arm. Eribulin dose was reduced according to protocol in six (40.0%) patients in the eribulin + AI arm and one (14.3%) patient in the eribulin arm.

### 3.4. Efficacy

An objective response was achieved in four (26.7%) patients randomized to the eribulin + AI arm (95% CI, 7.8–55.1%; *p* = 0.0541). In the eribulin arm, two (28.6%) patients had an objective response (95% CI, 3.7–71.0%). No complete responses were reported. The difference between the study arms was not significant (*p* = 0.918). Although the study met the statistical criteria to proceed with stage II, the exploratory combination did not reach a clinically meaningful improvement compared to eribulin monotherapy (Table 2).

No differences in CBR were observed; seven (46.7%) (95% CI, 21.3–73.4%) and three (42.9%) (95% CI, 9.9–81.6%) patients reached clinical benefit in the eribulin + AI and eribulin arms, respectively. The median DoR for patients treated with eribulin + AI was 3.6 months (IQR, 3.4–4.0 months), while DoR for patients treated with eribulin monotherapy was 6.9 months (IQR, 4.9–8.8 months). The median TTR was 1.9 (IQR, 1.8–2.5 months) and 2.1 (IQR, 2.1–2.1 months) months in the eribulin + AI and eribulin arms, respectively. The PFS rate at 6 months was 52.0% (95% CI, 22.3–75.2%) with eribulin + AI and 50.0% (95% CI, 11.1–80.4%) with eribulin monotherapy (hazard ratio [HR], 0.96; 95% CI, 0.2–3.9; *p* = 0.959) (Table 2).

At the time of this analysis, the OS was still immature with one (6.7%) death in the eribulin + AI arm and one (14.3%) death in the eribulin monotherapy arm. The OS rate at 12 months was 92.9% (95% CI, 59.1–99.0%) in the eribulin + AI arm and 80.0% (95% CI, 20.4–96.0%) in the eribulin monotherapy arm (HR, 0.84; 95% CI, 0.1–9.4; *p* = 0.888) (Supplementary Figure S1).

### 3.5. Subgroup Analyses

**Figure 2:**
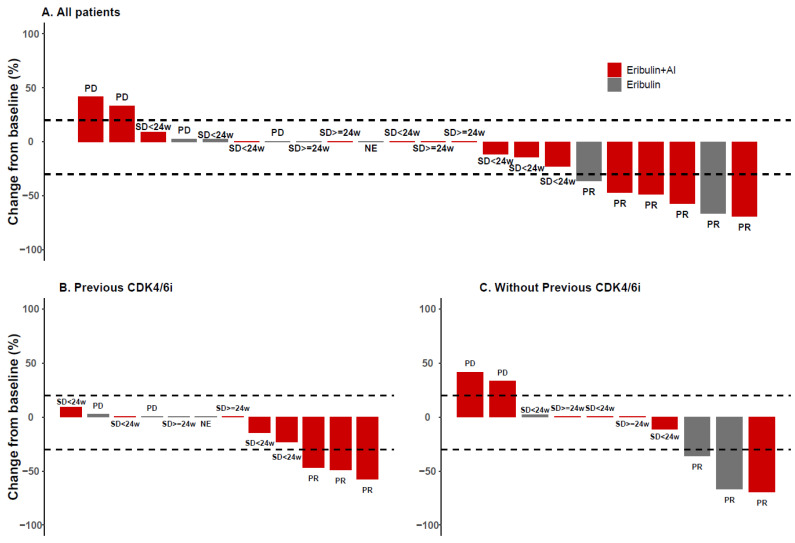
Overall response rate of patients who received eribulin + AI or eribulin only, according to prior treatment with CDK4/6i. (**A**) All patients, (**B**) CDK4/6I in previous line, (**C**) without previous CDK4/6i in previous line. AI, Aromatase inhibitors; CDK4/6i, Cyclin-dependent kinases 4 and 6 inhibitors; PD, Progressive disease; PR, Partial response; SD, Stable disease; NE, Not evaluable.

A significant interaction (*p* = 0.018) between the treatment arm and the previous CDK4/6i treatment was observed for ORR. For patients previously treated with CDK4/6i (N = 12), the difference in objective response was nonsignificant but numerically favorable to eribulin + AI. Specifically, three out of eight (37.5%) patients treated with eribulin + AI had an objective response, whereas none of the four patients treated with eribulin monotherapy achieved an objective response (*p* = 0.491). For patients without previous CDK4/6i treatment (N = 10), the difference in objective response was nonsignificant but numerically favorable to eribulin monotherapy with one out of seven (14.3%) patients and two out of three (66.7%) patients having an objective response after treatment with eribulin + AI and eribulin monotherapy, respectively (*p* = 0.183) (Figure 2). 

**Figure 3:**
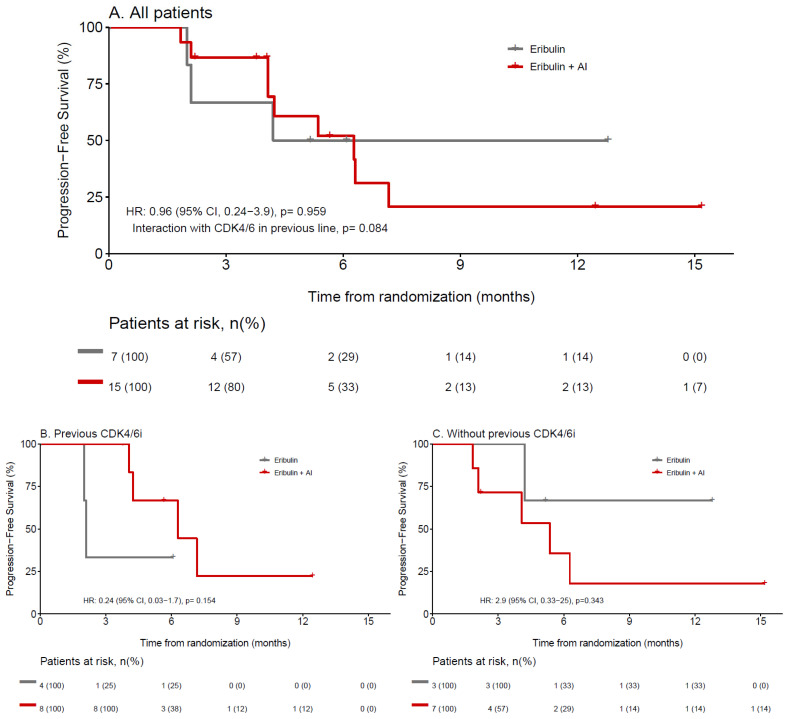
Progression-free survival of patients who received eribulin + AI or eribulin only, according to prior treatment with CDK4/6i. (**A**) All patients, (**B**) CDK4/6I in previous line, (**C**) without previous CDK4/6i in previous line. 95% CI, 95% confidence interval; AI, Aromatase inhibitor; CDK4/6i, Cyclin-dependent kinases 4 and 6 inhibitors; HR, Hazard ratio.

A trend interaction (*p* = 0.084) between the treatment arm and previous CDK4/6 inhibition was also observed for PFS. For patients with previous CDK4/6 inhibition, the difference in PFS was nonsignificant but numerically favorable to eribulin + AI. The PFS rate at 6 months was 66.7% (95% CI, 19.5–90.4%) with eribulin + AI and 33.3% (95% CI, 0.01–77.4%) with eribulin monotherapy (HR, 0.24; 95% CI, 0.03–1.7; *p* = 0.154). For patients without previous CDK4/6 inhibition, the difference in PFS was nonsignificant but numerically favorable to eribulin monotherapy. The PFS rate at 6 months was 35.7% (95% CI: 5.2–69.9%) with eribulin + AI and 66.7% (95% CI, 5.4–94.5%) with eribulin monotherapy (HR, 2.9; 95% CI, 0.3–25.0; *p* = 0.343) (Figure 3).

### 3.6. Safety

All patients experienced at least one AE. Eight (53.3%) and two (28.6%) patients had grade 3–4 AEs in the eribulin + AI and eribulin arms, respectively. The most common hematological AEs were neutropenia and anemia (Table 3). Grade 3–4 febrile neutropenia only occurred in one (14.3%) patient included in the eribulin arm.

The most common non-hematological AEs were fatigue, alopecia, and peripheral neuropathy (Table 3). The most common non-hematological grade 3–4 AEs were peripheral neuropathy and hepatotoxicity.

**Figure 4:**
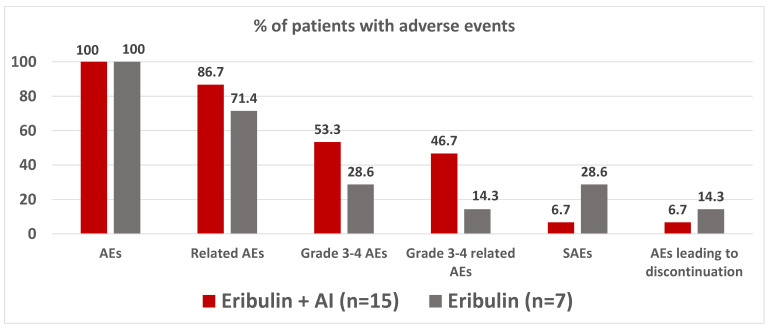
Summary of adverse events. AEs, Adverse events; AI, Aromatase inhibitor; SAEs, Serious adverse events.

Regarding the serious adverse events (SAEs), one (6.7%) patient experienced an SAE in the eribulin + AI arm (grade 3 chest pain related to breast cancer) and two (28.6%) patients experienced an SAE in the eribulin arm (grade 3 pulmonary embolism and grade 3 pneumonitis). All SAEs were unrelated to the study treatment (Figure 4). Permanent discontinuation of study treatment due to AEs occurred in one (6.7%) patient in the eribulin + AI arm (grade 3 peripheral neuropathy) and one (14.3%) patient in the eribulin arm (grade 3 pneumonitis). No treatment-related deaths were reported (Figure 4).

## 4. Discussion

Despite a better knowledge of the molecular biology of luminal ABC and the introduction of several targeted therapies that clearly improve ET efficacy (CDK4/6i, mammalian target of rapamycin [mTOR] inhibitors, and phosphatidylinositol 3-kinase [PI3K] inhibitors), all patients who initially respond to ET become finally resistant to this treatment [2]. For this reason, the development of new endocrine treatments, such as selective estrogen receptor degraders (SERDs), or therapeutic strategies that restore endocrine sensitivity is critical.

In this context, the REVERT study was designed with the aim of restoring endocrine sensitivity through the combination of eribulin and AI in AI-resistant luminal ABC patients. Although eribulin is widely used in patients with previously treated ABC, data regarding its antitumor activity using a chemo–endocrine therapeutic approach are not available.

The addition of an AI to eribulin did not improve outcomes in the REVERT study and, therefore, our study failed to demonstrate a possible effect of the combination of eribulin and AI in restoring endocrine sensitivity, despite the promising biological evidence that was observed in the NeoEribulin-SOLTI1007 study [12]. This trial showed that 44.44% of tumors with a luminal B molecular subtype at baseline developed characteristics consistent with the less aggressive luminal A molecular subtype after four cycles of neoadjuvant treatment with eribulin. This phenotypic change was attributed to the increased expression of luminal-related genes (e.g., *ESR1* and *NAT1*), negative regulation of apoptosis (e.g., *BCL2* and *IL6*) and angiogenesis (e.g., *ANGPTL4* and *HIF1A*), and a decrease in the expression of cell cycle-related genes (e.g., *CCNB1*, *RAD17*, and *MKI67*) and genes related to microtubule cytoskeleton organization (e.g., *AURKA*, *CENPA*, and *KIF23*) [12].

Patients with luminal ABC and previously treated with CDK4/6i represent a population of special interest since very few treatments have been explored in this subgroup of patients. The antitumor activity of classical endocrine drugs as single agents is limited, with a median PFS of approximately two months. For this reason, the combination of ET with PI3K/mTOR inhibitors, everolimus or alpelisib according to *PIK3CA* mutational status, therapeutic strategies based on CDK4/6i rechallenge, or even chemotherapy-containing regimens, are commonly used options in this scenario [11,13,14,15]. Interestingly, in the REVERT study, a significant interaction between the treatment arm and previous CDK4/6i treatment was observed for ORR and PFS, suggesting that ET could improve the antitumor efficacy of eribulin in luminal ABC patients refractory to CDK4/6i. In this way, a retrospective observational study that examined the safety and effectiveness of eribulin in this population demonstrated that eribulin might be a potential treatment option following prior treatment with CDK4/6i [16]. In addition, eribulin has shown superior antitumor activity to capecitabine after fulvestrant plus palbociclib in preclinical models [17].

EMT has been associated with acquired resistance to CDK4/6i through the activation of the transforming growth factor beta (TGF-β) pathway. Canonical intracellular TGF-β signal transduction occurs through the Smad pathway. This involves a type I receptor-induced phosphorylation of receptor-regulated Smads 2 and 3 (R-Smad2–3), which associate with the common mediator Smad4, forming heteromeric complexes that induce the activation of EMT transcription factors [18]. One of the non-mitotic effects of eribulin is the reversal of this EMT, decreasing the expression of mesenchymal marker genes, while increasing the expression of epithelial markers and in consequence, reducing migration and invasiveness capabilities [7]. This mechanism of action of eribulin could be responsible for the higher efficacy observed with eribulin in patients previously treated with CDK4/6i.

Hence, REVERT results open the door to exploring the efficacy of eribulin in combination with ET in patients who have progressed to a previous CDK4/6i, mainly with new endocrine agents, including novel SERDs. The identification of predictors of response to this concurrent chemo–endocrine therapy will be critical to identify those patients that are more likely to benefit from this strategy.

Overall, the toxicity profile was consistent with the known safety profile of eribulin, with an unexpectedly higher incidence of grade 3–4 AEs in patients treated with eribulin + AI.

The main limitations of this study are the small sample size and the premature study closure. Nevertheless, these results provide support for the additional investigation of this chemo–endocrine therapeutic approach in AI-resistant luminal ABC patients refractory to CDK4/6i in larger randomized, controlled trials.

In conclusion, the combination of eribulin plus an AI does not seem to improve ORR, PFS, or OS compared with eribulin monotherapy in patients with AI-resistant luminal ABC. A significant interaction in favor of eribulin + AI was observed for patients resistant to CDK4/6i with a numerically higher ORR and PFS. Eribulin plus ET deserves further investigation in patients with AI/CDK4/6i-resistant HR[+]/HER2[−] ABC.

## Figures and Tables

**Table 1:** Patients’ characteristics.

Characteristics, *n* (%)	All Patients (*n* = 22)	Eribulin + AI (*n* = 15)	Eribulin (*n* = 7)
**Age in years, median (range)**	65 (56–77)	60 (50–77)	63 (50–77)
**Race**			
Black	2 (9.1)	1 (6.7)	1 (14.3)
Hispanic or Latino	2 (9.1)	2 (13.3)	0 (0.0)
White	18 (82.8)	12 (80.0)	6 (85.7)
**ECOG PS score**			
0	18 (81.8)	13 (86.7)	5 (71.4)
1	4 (18.2)	2 (13.3)	2 (28.6)
**Visceral involvement**			
Yes	22 (100)	15 (100)	7 (100)
**Liver involvement**			
Yes	14 (63.6)	10 (66.7)	4 (57.1)
**Number of metastatic sites**			
<3	14 (63.6)	10 (66.7)	4 (57.1)
≥3	8 (36.4)	5 (33.3)	3 (42.9)
**Prior lines of ET for ABC**			
0	9 (40.9)	6 (40.0)	3 (42.9)
1	7 (31.8)	5 (33.3)	2 (28.6)
2	5 (22.7)	3 (20.0)	2 (28.6)
3	1 (4.5)	1 (6.7)	0 (0.0)
**AI administered in the last regimen**			
Letrozole	15 (68.2)	10 (66.7)	5 (71.4)
Exemestane	6 (27.3)	5 (33.3)	1 (14.3)
Anastrozole	1 (4.5)	0(0.0)	1 (14.3)
**Previous treatment with CDK4/6i**			
Yes	12 (54.5)	8 (53.3)	4 (57.1)
No	10 (45.5)	7 (46.7)	3 (42.9)
**Treatment with CDK4/6i in the immediate line of therapy**			
Yes	9 (40.9)	6 (40.0)	3 (42.9)
No	13 (59.1)	9 (60.0)	4 (57.1)

ABC, advanced breast cancer; AI, Aromatase inhibitor; CDK4/6, cyclin-dependent kinases 4 and 6; ECOG: Eastern Cooperative Oncology Group; ET, Endocrine therapy; PS, Performance status.

**Table 2:** Efficacy endpoint results.

	Eribulin + AI (N = 15)	Eribulin(N = 7)	*p*
**Overall response rate, (95% CI)**	26.7 (7.8–55.1) *p* = 0.0541	28.6 (3.7–71.0)	0.918
**Best response, *n* (%)**			-
Complete response	0 (0.0)	0 (0.0)	
Partial response	4 (26.7)	2 (28.6)	
Stable disease ≥ 24 weeks	3 (20.0)	1 (14.3)	
Stable disease < 24 weeks	6 (40.0)	1 (14.3)	
Progressive disease	2 (13.3)	2 (28.6)	
Not evaluable	0 (0.0)	1 (0.0)	
**Clinical benefit rate, (95% CI)**	46.7% (21.3–73.4%)	42.9% (9.9–81.6%)	0.878
Median duration of response, months (IQR) *	3.6 (3.4–4.0)	6.9 (4.9–8.8)	0.800
Median time to response, months (IQR) *	1.9 (1.8–2.5)	2.1 (2.1–2.1)	0.481
**Progression-free survival rate at 6 months (95% CI)**	52.0% (22.3–75.2%)	50.0% (11.1–80.4%)	0.959
**Overall survival rate at 12 months (95% CI)**	92.9% (59.1–99.0%)	80.0% (20.4–96.0%)	0.888

* Time to response and duration of response were analyzed in responders. 95% CI, 95% confidence interval; AI, Aromatase inhibitor; IQR, Interquartile range (percentile 25–percentile 75).

**Table 3:** Adverse events with an incidence of at least 14% and/or grade ≥ 3 in the safety population.

	Eribulin + AI		Eribulin	
	Any Grade	Grade 3–4	Any Grade	Grade 3–4
**All AEs**	15 (100.0%)	8 (53.3%)	7 (100.0%)	2 (28.6%)
**Hematological**	8 (53.3%)	4 (26.7%)	2 (28.6%)	1 (4.3%)
Neutropenia	5 (33.3%)	4 (26.7%)	2 (28.6%)	1 (14.3%)
Anemia	3 (20.0%)	0 (0.0%)	1 (14.3%)	0 (0.0%)
Febrile neutropenia	0 (0.0%)	0 (0.0%)	1 (14.3%)	1 (14.3%)
Leukopenia	1 (6.7%)	0 (0.0%)	1 (14.3%)	0 (0.0%)
Thrombocytopenia	0 (0.0%)	0 (0.0%)	1 (14.3%)	0 (0.0%)
**Non-hematological**	15 (100.0%)	6 (40.0%)	7 (100.0%)	2 (28.6%)
Fatigue	6 (40.0%)	0 (0.0%)	2 (28.6%)	0 (0.0%)
Peripheral neuropathy	5 (33.3%)	2 (13.3%)	1 (14.3%)	0 (0.0%)
Nausea	4 (26.7%)	0 (0.0%)	0 (0.0%)	0 (0.0%)
Hepatotoxicity	3 (20.0%)	2 (13.3%)	1 (14.3%)	1 (14.3%)
Alopecia	3 (20.0%)	0 (0%)	3 (42.9%)	0 (0.0%)
Headache	3 (20.0%)	0 (0.0%)	0 (0.0%)	0 (0.0%)
Pyrexia	3 (20.0%)	0 (0.0%)	0 (0.0%)	0 (0.0%)
Chest pain	2 (13.3%)	1 (6.7%)	1 (14.3%)	0 (0.0%)
Pneumonitis	0 (0.0%)	0 (0.0%)	1 (14.3%)	1 (14.3%)
Pulmonary embolism	0 (0.0%)	0 (0.0%)	1 (14.3%)	1 (14.3%)

AEs, Adverse events; AI, Aromatase inhibitor.

## Data Availability

The datasets generated and/or analyzed during the current study are not publicly available due to risk of personal information leakage but are available from the corresponding author on rea-sonable request.

## Supplementary Materials

The following supporting information can be downloaded at: https://www.mdpi.com/article/10.3390/cancers14235880/s1, Figure S1: Overall survival by treatment arm. 95% CI, 95% confidence interval; AI, Aromatase inhibitor; HR, Hazard ratio.
