# Supplementary material for: Challenging Endocrine Sensitivity of Hormone Receptor-Positive/HER2-Negative Advanced Breast Cancer with the Combination of Eribulin and Endocrine Therapy: The REVERT Study
Source: Cancers (Basel). 2022 Nov 29;14(23):5880. doi: 10.3390/cancers14235880 (PMC9737152; doi:10.3390/cancers14235880)
Supplement: Supplementary file 1 [file cancers-14-05880-s001.zip › cancers-2052410-supplementary.pdf]

cancers

MDPI

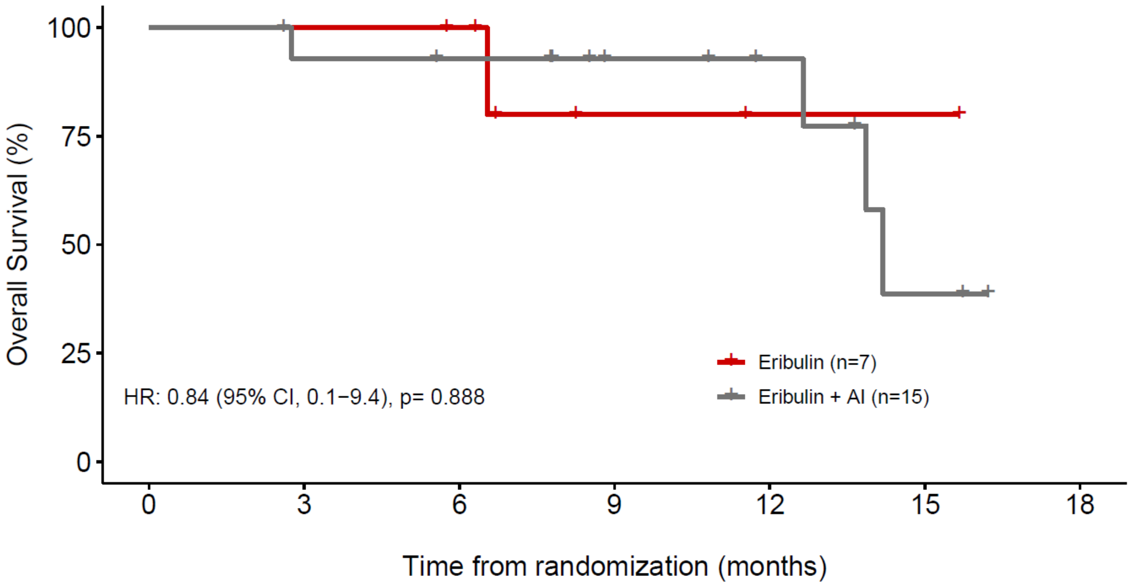

Patients at risk, n(%)

Eribulin (red)	7 (100)	7 (100)	6 (86)	2 (29)	1 (14)	1 (14)	0 (0)
Eribulin + AI (grey)	15 (100)	13 (87)	12 (80)	8 (53)	6 (40)	2 (13)	0 (0)

Supplementary Figure S1. Overall survival by treatment arm. 95% CI, 95% confidence interval; AI, Aromatase inhibitor; HR, Hazard ratio.
